# Machine learning outperforms traditional logistic regression and offers new possibilities for cardiovascular risk prediction: A study involving 143,043 Chinese patients with hypertension

**DOI:** 10.3389/fcvm.2022.1025705

**Published:** 2022-11-14

**Authors:** Yang Xi, Hongyi Wang, Ningling Sun

**Affiliations:** Department of Hypertension, Peking University People’s Hospital, Beijing, China

**Keywords:** machine learning, CVD, risk prediction, hypertension, traditional logistic regression

## Abstract

**Introduction:**

Identifying people at risk of cardiovascular diseases (CVD) is a cornerstone of preventive cardiology. We developed machine learning (ML) algorithms and investigated their performance in predicting patients’ current CVD risk (coronary heart disease and stroke in this study).

**Materials and methods:**

We compared traditional logistic regression (LR) with five ML algorithms LR with Elastic-Net, Random Forest (RF), XGBoost (XGB), Support Vector Machine, Deep Learning, and an Ensemble model averaging predictions from RF, XGB, and Deep Learning for CVD risk prediction using pre-existing patient-level data from a multi-center, cross-sectional study (the Microalbuminuria Screening in Hypertensive Patients Project initiated by the China International Exchange and Promotive Association for Medical and Healthcare) that enrolled 143,043 patients with hypertension from 600 tertiary, secondary, or community hospitals. Each of the five ML algorithms incorporated 18 variables, such as demographics, examinations, comorbidities, and treatment regimens, and were trained and evaluated using 5-fold cross-validation. Predictive accuracy was assessed by the area under the receiver operating curve (AUROC).

**Results:**

Patients’ mean age was 62 ± 12 years and 57% were men. Advanced ML algorithms outperformed the traditional LR model. Particularly, the Ensemble model had superior discrimination with an AUROC of 0.760 than LR (AUC = 0.737) and other tested models.

**Conclusion:**

We establishes an Ensemble model that shows better performance in predicting patients’ current CVD risk using routine information compared to the traditional LR model. ML can help physicians design follow-up plans with more accurate results, offering new possibilities for short-term risk prediction and early detection. Further, ML models can be trained with longitudinal data and used to predict long-term CVD risks, thereby informing CVD prevention.

## Introduction

Cardiovascular disease (CVD) is the leading burden of disease in China, with a prevalence of approximately one in five adults and accounting for more than 40% of the total deaths (1, 2). According to the latest CVD report in 2018, there were 290 million CVD patients in China, including 1.3 million cases of stroke and 1.1 million cases of coronary heart disease (CHD) (1).

Cardiovascular disease (CVDs) is highly preventable—it was estimated that up to 90% of CVDs could be prevented (3, 4). Early prevention and screening for high-risk populations are key strategies for reducing the burden of CVDs (3, 5). At the individual level, some risk factors for CVDs have been well-established, such as smoking, blood pressure, diabetes and obesity, air pollution, and social determinants including health system and health policies (6). As early as 1996, the concept of matching the intensity of risk factor management to the hazard of CVDs was first proposed (7). Currently, there is an increasing emphasis on stratifying the risk of CVDs to guide the prevention and treatment schedules (8–10).

Several CVD risk assessment tools have been developed from different populations, such as the American College of Cardiology/American Heart Association atherosclerotic cardiovascular disease (ASCVD) pooled cohort equations, the Framingham Risk Score, the Systematic Coronary Risk Evaluation in Europe, the Global Registry of Acute Coronary Events acute coronary syndrome (ACS) risk and mortality calculator, the Thrombolysis In Myocardial Infarction Risk Score, and the QRISK in the United Kingdom and Scottish ASSIGN risk score (11–16). The applicability of these tools in China, however, is limited by the fact that they originated from Western countries, where the disease pattern of CVDs may substantially differ from that of China. Currently, Chinese guidelines recommend a simplified risk scoring table with common predictors including age groups, low-density lipoprotein cholesterol (LDL-C) or total cholesterol (TC), smoking, body mass index (BMI), blood pressure, hypertension, and diabetes mellitus (DM) to predict ASCVD risks (10). Some other CVD risk prediction tools are also available for the Chinese population, such as the in-hospital mortality risk prediction tool for ACS patients, the 10-year ASCVD risk prediction tool from the China-PAR Project, the CVD-death risk prediction tool, and the 5-year CVD risk prediction tool for patients with DM (17–20). To date, there is no prediction tool specific to Chinese patients with hypertension and the accuracy of CVD risk assessment remains an issue of concern (21, 22).

Machine learning (ML), a technique that allows computer systems to learn from data and effectively perform a specific task without explicit instructions, offers an alternative approach to predict an individual’s CVD risk (23). Previous studies have demonstrated that ML can significantly improve the model performance and the accuracy of the CVD risk prediction (24, 25). Given the present availability of individual-level data in China, ML is also expected to improve the CVD risk prediction for the Chinese hypertensive population.

On this basis, our study aimed to explore the potential of using ML to predict CVD risk for the Chinese hypertensive population based on the information routinely collected in clinical settings and to evaluate the performance of each ML model.

## Materials and methods

### Data source

**FIGURE 1:**
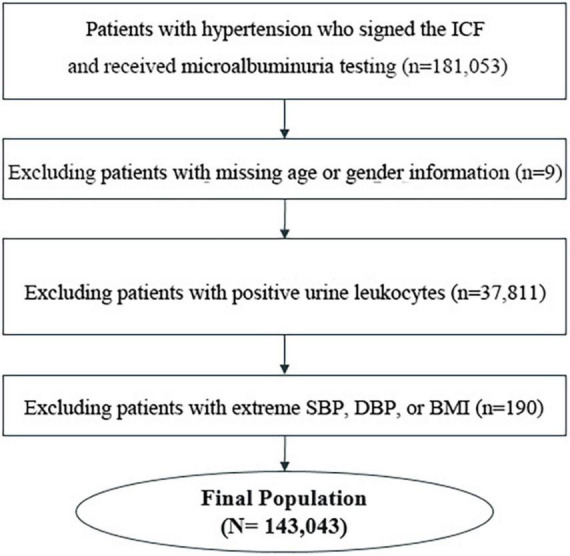
Flow diagram showing numbers of included and excluded patients.

The study dataset was based on the Microalbuminuria Screening in Hypertensive Patients Project, a multicenter, cross-sectional study initiated by the China International Exchange and Promotive Association for Medical and Healthcare. A total of 143,043 inpatients and outpatients with hypertension from 600 tertiary, secondary, or community hospitals in China from November 2016 to August 2017 were included in the analysis. The flowchart of patient selection is shown in Figure 1. The project was carried out in accordance with the Good Pharmacoepidemiology Practice. All patients signed informed consent before participation. The study was approved by the Ethical Committee of Peking University People’s Hospital [2013–17].

### Variables

Eighteen variables were included to develop the models, including demographics (gender, age, and geographic information), health indicators [body mass index (BMI), systolic blood pressure (SBP), diastolic blood pressure (DBP), carotid artery thickening, left ventricular hypertrophy, history of smoking, and family history of hypertension], results of laboratory tests [levels of low-density lipoprotein cholesterol (LDL-C) and urine albumin to creatinine ratio (UACR)], current treatment regimen, type of visit (outpatient or inpatient), whether hypertension was newly diagnosed, whether UACR was newly detected, presence of diabetes, and length of use of renin-angiotensin system inhibitor (RASI). Categorical variables were further converted into dummy variables. Microalbuminuria was defined as UACR between 30 and 300 mg/g.

### Machine learning algorithms

The primary outcome was CVD events defined as the development of CHD and/or stroke. To identify the best model performance, a total of six machine learning methods were compared, including the Logistic Regression (LR) with Elastic-Net, Random Forest, XGBoost, Support Vector Machine, Deep Learning, and Ensemble models. The LR model was used as the benchmark reference. These algorithms were selected based on the ease of implementation into current datasets.

Each algorithm was trained and evaluated using 5-fold cross-validation. Specifically, the final data sample was randomly stratified and split into the modeling dataset (80%) and the hold-out dataset (20%). Then the modeling dataset was randomly stratified into five evaluation folds/samples of equal size: one evaluation fold as the test dataset and the other four evaluation folds as the training dataset. The area under receiver operating characteristic curves (AUROCs) and area under precision-recall curves (AUPRCs) were used to evaluate the performance of the algorithms.

The hold-out dataset was used to evaluate the optimal threshold for predicting short-term CVD events in patients with hypertension. Two strategies, respectively, from a clinical perspective and a data science perspective, were used to explore the effect of prediction threshold values on false positive/negative cases and rates. We chose three clinical thresholds, 0.05 (T1), 0.10 (T2), and 0.15 (T3), based on the high-risk rating for ASCVD within 10 years from the 2016 Chinese Adult Dyslipidemia Prevention and Treatment Guideline (26) and the extreme high-risk rating of ASCVD from the 2013 ACC/AHA Guideline on the Assessment of Cardiovascular Risk (27) and the European SCORE (28). Two additional thresholds were chosen from a data science perspective—the first was set to fix the sensitivity at 90% (T4); the second was ROC01, which was designed to minimize the distance between the ROC plot and the point (0,1) to balance sensitivity and specificity (T5).

All analyses were performed using Python 3.6. *P* values of less than 0.05 were considered statistically significant.

## Results

### Study population characteristics

Briefly, a total of 143,043 patients were included in the analysis, of whom 26.2% were newly diagnosed cases, 30.1% controlled their blood pressure by treatment, and 21.6% experienced at least one CVD event. The patient characteristics are shown in Table 1.

**TABLE 1:** Characteristics of included patients.

Characteristic	All patients (*N* = 143,043)	CVD patients (*N* = 30,903)	Non-CVD patients (*N* = 112,140)	*P*-value
Age (years), mean ± sd	61.8 ± 12.3	65.7 ± 12.1	60.7 ± 12.2	< 0.001
**Gender, n (%)**				
Male	81,356 (56.9%)	18,301 (59.2%)	63,055 (56.2%)	< 0.001
Female	61,687 (43.1%)	12,602 (40.8%)	49,085 (43.8%)	
**Area, n (%)**				
Central south	18,970 (13.3%)	4,165 (13.5%)	14,805 (13.2%)	< 0.001
North	21,714 (15.2%)	7,067 (22.9%)	14,647 (13.1%)	
East	74,044 (51.8%)	12,562 (40.6%)	61,482 (54.8%)	
Southwest	17,976 (12.6%)	3,885 (12.6%)	14,091 (12.6%)	
Northwest	5,363 (3.7%)	1,604 (5.2%)	3,759 (3.4%)	
Northeast	4,976 (3.5%)	1,620 (5.2%)	3,356 (3.0%)	
**Type of visit, n (%)**				
Outpatient	94,233 (67.6%)	12,476 (41.5%)	81,757 (74.8%)	< 0.001
Inpatient	45,063 (32.4%)	17,591 (58.5%)	27,472 (25.2%)	
**BMI (kg/m** ^2^ **), n (%)**				
Underweight	3,656 (2.6%)	823 (2.7%)	2,833 (2.5%)	< 0.001
Normal	67,952 (47.5%)	13,577 (43.9%)	54,375 (48.5%)	
Overweight	57,636 (40.3%)	12,959 (41.9%)	44,677 (39.8%)	
Obesity	13,799 (9.6%)	3,544 (11.5%)	10,255 (9.1%)	
**SBP (mmHg)**	141.9 ± 18.2	142.3 ± 19.8	141.8 ± 17.7	< 0.001
**DBP (mmHg)**	86.1 ± 13.4	84.9 ± 14.2	86.4 ± 13.1	< 0.001
**Newly diagnosed hypertension, n (%)**				
Yes	37,536 (26.2%)	7,130 (23.1%)	30,406 (27.1%)	< 0.001
No	105,507 (73.8%)	23,773 (76.9%)	81,734 (72.9%)	
**Carotid artery thickening, n (%)**				
Yes	23,073 (16.1%)	8,116 (26.3%)	14,957 (13.3%)	< 0.001
No	119,970 (83.9%)	22,787 (73.7%)	97,183 (86.7%)	
**Left ventricular hypertrophy, n (%)**				
Yes	20,404 (14.3%)	6,242 (20.2%)	14,162 (12.6%)	< 0.001
No	122,639 (85.7%)	24,661 (79.8%)	97,978 (87.4%)	
**History of smoking, n (%)**				
Yes	24,922 (17.4%)	7,119 (23.0%)	17,803 (15.9%)	< 0.001
No	118,121 (82.6%)	23,784 (78.8%)	94,337 (84.1%)	
**Family history of hypertension, n (%)**				
Yes	29,937 (20.9%)	6,560 (21.2%)	23,377 (20.8%)	0.14
No	113,106 (79.1%)	24,343 (78.8%)	88,763 (79.2%)	
**LDL-C level (mmol/L), n (%)**				
< 1.8	7,782 (23.3%)	2,429 (21.9%)	5,353 (23.9%)	< 0.001
1.8 ≤ LDL-C < 2.6	12,964 (38.7%)	4,160 (37.5%)	8,804 (39.4%)	
2.6 ≤ LDL-C < 3.4	9,314 (27.8%)	3,147 (28.3%)	6,167 (27.6%)	
≥ 3.4	3,410 (10.2%)	1,371 (12.3%)	2,039 (9.1%)	
**UACR, n**				
Normal	50,950 (35.6%)	10,383 (33.6%)	40,567 (36.2%)	< 0.001
Microalbuminuria	90,204 (63.1%)	20,107 (65.1%)	70,097 (62.5%)	
Albuminuria	1,889 (1.3%)	413 (1.3%)	1,476 (1.3%)	
**Newly detected UACR, n (%)**				
Yes	107,571 (82.5%)	22876 (82.4%)	84695 (82.5%)	0.74
No	22,892 (17.5%)	4891 (17.6%)	18001 (17.5%)	
**Diabetes, n (%)**				
Yes	33,161 (23.2%)	6,129 (19.8%)	27,032 (24.1%)	< 0.001
No	109,882 (76.8%)	24,774 (80.2%)	85,108 (75.9%)	
**Current antihypertensive regimen, n (%)**				
Diuretics only	6,728 (6.0%)	808 (3.3%)	5,920 (6.8%)	< 0.001
β receptor blockers only	11,912 (10.7%)	2,202 (8.9%)	9,710 (11.2%)	
CCBs only	20,766 (18.6%)	3,291 (13.3%)	17,475 (20.1%)	
ACEIs only	10,393 (9.3%)	2,118 (8.6%)	8,275 (9.5%)	
ARB normal dosage only	12,745 (11.4%)	2,011 (8.2%)	10,734 (12.3%)	
ARB double dosage only	794 (0.7%)	147 (0.6%)	647 (0.7%)	
ARBs + Diuretics	9,550 (8.6%)	1,952 (7.9%)	7,598 (8.7%)	
ARBs + CCBs	10,458 (9.4%)	2,324 (9.4%)	8,134 (9.3%)	
Other combination of two drugs	18,958 (17.0%)	5,828 (23.6%)	13,130 (15.1%)	
Other combinations	9,366 (8.4%)	3,977 (16.1%)	5,389 (6.2%)	
**Use of RASI, n (%)**				
< 2 years	58,771 (60.3%)	11,466 (53.7%)	47,305 (62.2%)	< 0.001
≥ 2 years	38,629 (39.7%)	9,876 (46.3%)	28,753 (37.8%)	

BMI, body mass index; SBP, systolic blood pressure; DBP, diastolic blood pressure; LDL-C, low-density lipoprotein cholesterol; UACR, urine albumin to creatinine ratio; CCB, calcium channel blocker; ACEI, angiotensin-converting enzyme inhibitor; ARB, angiotensin II receptor blocker; RASI, renin-angiotensin system inhibitor. *P*-values were referred to the comparison between the CVD and non-CVD groups.

### Model performance comparison

**FIGURE 2:**
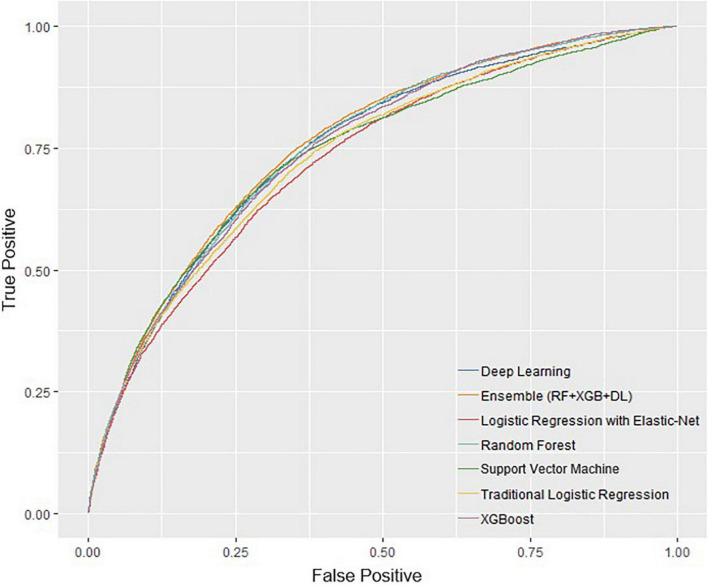
Receiver operating characteristic curves of all models.

**FIGURE 3:**
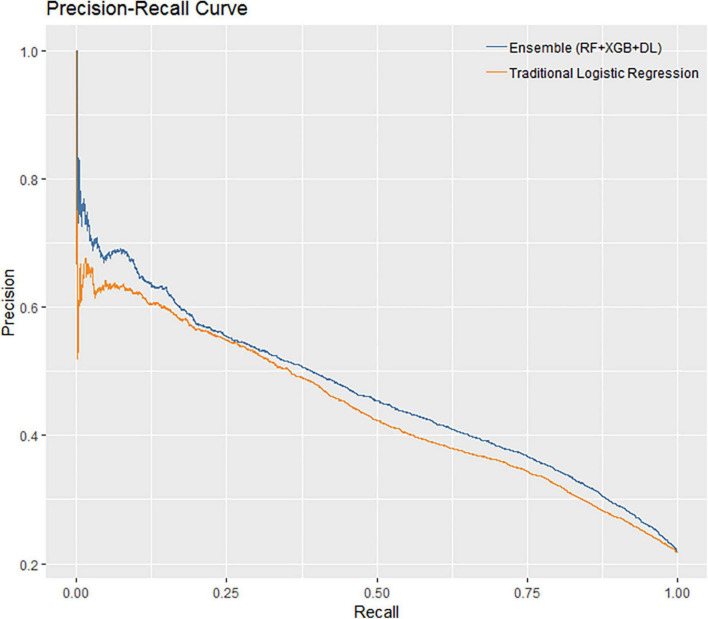
Precision-recall curves of ensemble model and traditional logistic regression model.

The performance of all algorithms was reasonably good (Figure 2 and Table 2), indicated by AUROCs between 0.7 and 0.8. The Ensemble model was the best performing model with the highest AUROC, compared with the LR model [Ensemble model: AUROC = 0.760, 95% confidence interval (CI): 0.754–0.766]. The machine learning models generally outperformed the LR model (Figure 2 and Table 2). However, the LR with Elastic-Net model showed poorer performance compared to the LR model (LR: AUROC = 0.737, 95% CI: 0.730–0.742; LR with Elastic-Net: AUROC = 0.728, 95% CI: 0.723–0.733). The Ensemble model was also the best performing model based on AUPRC (AUPRC = 0.465, 95% CI: 0.454–0.475, Figure 3 and Table 3).

**TABLE 2:** Model performance measured by area under receiver operating characteristic curve.

Model	AUROC	SE	95% CI		Δ^1^	*P*-value
			LCL	UCL		
Logistic regression (Benchmark)	0.737	0.001	0.730	0.742	-	-
Logistic regression with elastic-net	0.728	0.001	0.723	0.733	−0.9%	< 0.001
Support vector machine	0.740	0.001	0.735	0.745	0.3%	0.072
Random forest	0.754	0.001	0.747	0.761	1.7%	< 0.001
XGBoost	0.751	0.001	0.743	0.756	1.4%	< 0.001
Deep learning	0.750	0.001	0.746	0.755	1.3%	< 0.001
Ensemble	0.760	0.001	0.754	0.766	2.3%	< 0.001

^1^Δ refers to absolute changes from benchmark.

AUROC, area under receiver operating characteristic curve; SE, standard error; CI, confidence interval; LCL, lower confidence limit; UCL, upper confidence limit.

**TABLE 3:** Model performance measured by area under precision-recall curves.

Model	AUPRC	SE	95% CI		Δ ^1^	*P*-value
			LCL	UCL		
Logistic regression (Benchmark)	0.439	0.002	0.427	0.450	-	-
Logistic regression with elastic-net	0.426	0.002	0.415	0.436	−1.3%	< 0.001
Random forest	0.460	0.002	0.449	0.471	2.0%	< 0.001
XGBoost	0.448	0.002	0.436	0.456	0.8%	0.003
Deep learning	0.454	0.002	0.442	0.465	1.5%	< 0.001
Ensemble	0.465	0.002	0.454	0.475	2.6%	< 0.001

^1^Δ refers to absolute changes from benchmark.

AUPRC, area under the precision-recall curve; SE, standard error; CI, confidence interval; LCL, lower confidence limit; UCL, upper confidence limit.

### Error and sensitivity analysis of the ensemble model

**FIGURE 4:**
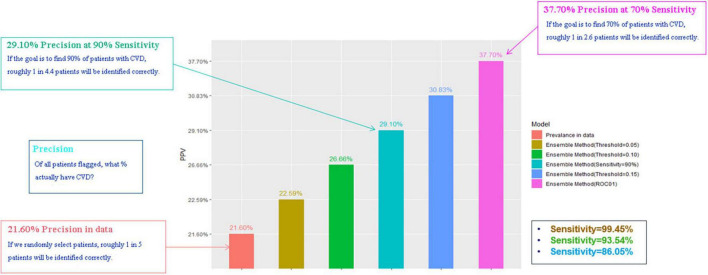
Precision based on different thresholds.

We compared the Ensemble model with the LR model to determine the optimal threshold for classifying patients as likely or unlikely to develop CVDs in a short term. Table 4 shows the results of altering the threshold on false positive rates and false negative rates. T1 was the most sensitive threshold where the Ensemble model incorrectly classified 32 patients as low risk of developing CVDs (LR = 99.03%; Ensemble = 99.48%). The most specific threshold was T5 where the Ensemble model misclassified 7,075 patients as high-risk (LR = 65.84%; Ensemble = 68.45%). Of all the thresholds tested, T5 showed the best performance because it misclassified the least number of patients (LR = 9,505; Ensemble = 8,995), compared with T1 which misclassified the greatest number of patients (LR = 21,259; Ensemble = 21,100). Overall, using T5 on the Ensemble model showed the best precision, with correct identification of 37.7% of patients (Figure 4). However, this 8.6% increase in precision of using T5 over T4 came at a 20% reduction in sensitivity.

**TABLE 4:** Sensitivity analysis of Ensemble model and its comparison with benchmark.

	Thre = 0.05	Δ^1^	Thre = 0.1	Δ^1^	Thre = 0.15	Δ^1^	Sens = 90%	Δ^1^	ROC01	Δ^1^
**False positive case**										
Benchmark (LR)	21,199	-	15,061	-	10,383	-	14,861	-	7,662	-
Ensemble method	21,068	−131	15,980	919	12,102	1719	13,551	−1310	7,075	−587
**False positive rate** ^2^										
Benchmark (LR)	94.52%	-	67.15%	-	46.29%	-	66.26%	-	34.16%	-
Ensemble method	93.94%	−0.58%	71.25%	4.10%	53.96%	7.66%	60.42%	−5.84%	31.55%	−2.62%
**False negative case**										
Benchmark (LR)	60	-	586	-	1,238	-	618	-	1,843	-
Ensemble method	32	−28	373	−213	788	−450	618	0	1,920	77
**False negative rate** ^3^										
Benchmark (LR)	0.97%	-	9.48%	-	20.03%	-	10.00%	-	29.82%	-
Ensemble method	0.52%	−0.45%	6.03%	6.03%	12.75%	−7.28%	10.00%	0	31.06%	1.25%

^1^Δ refers to absolute changes from benchmark.

^2^False Positive Rate = False Positive Cases/(False Positive Cases + True Negative Patients) = 1-Specificity.

^3^False Negative Rate = False Negative Cases/(False Negative Cases + True Positive Patients) = 1-Sensitivity.

LR: traditional logistic regression model.

## Discussion

Although CVDs is the leading burden of disease in China that affects 290 million people (1, 2), up to 90% of CVDs can be prevented (3–5). There is an increasing emphasis on risk stratification for CVDs to guide the prevention and treatment schedules (8–10).

This study is a cross-sectional survey of hypertensive patients with or without CVDs. In this study, we established ML models for predicting CVD risk based on a large-sample database with comprehensive clinical information. The ML models, except for LR with Elastic-Net model, all showed better performance than traditional LR, as measured by both AUROC and AUPRC. Since the AUROCs are between 0.7 and 0.8, the performance of all models can be considered reasonably good. In addition to AUROC, we also calculated AUPRC as the model performance measure. In our data, 112,140 (78.4%) patients were non-CVD as negative cases. AUPRC is, therefore, suitable to measure the model performance. Both AUROC and AUPRC showed that the Ensemble model was the best performing model.

A low threshold can help identify as many patients as possible who would develop CVDs in a short term. In contrast, the optimal T5 threshold will reduce misclassification and minimize patients’ overall anxiety about future risk. In practice, which threshold should be chosen also depends on physician preference.

Previous CVD risk assessment models were mainly based on the simplest parameters, such as blood pressure, age, lipids, BMI, and drug treatment. The atherosclerotic cardiovascular disease (ASCVD) cohort equation of ACC/AHA in the United States, the Framingham risk score, the European Systemic coronary risk assessment model (10–16), the 10-year ASCVD risk prediction tool of the PAR project in China, and the CVD death risk prediction tool (17–20) are all in this case. Compared with the previous models, our machine learning prediction model adds some new parameters commonly detected in patients with hypertension at present, including blood glucose, creatinine, microalbuminuria, and carotid intima-media thickening, so our model has good availability and can provide more accurate risk predictions of CVD. In addition, the present study was based on a large-sample cross-sectional investigation, which provided sufficient statistical power for the construction of the machine learning model.

There are several limitations in this study, mainly due to issues related to data quality. First, the number of variables collected at baseline was limited. Only eighteen variables were collected by the survey, which may not cover all risk factors associated with CVDs. Nevertheless, our models with these variables showed reasonably good performance indicated by AUROC over 0.7. Second, some variables were not clearly defined during data collection. For example, in terms of the blood pressure readings, we were unable to determine whether the measurements were before or after medication, which may cause heterogeneity and affect the accuracy of the effect estimates. Thirdly, several key variables were collected through open-ended questions. This resulted in a high rate of missing values. For example, nearly 90% of participants did not provide valid information on the duration of hypertension.

Our model can be adopted in healthcare settings where key clinical information is available. In future work, we will improve the model by collecting additional information related to cardiovascular risk and comparing it with other models being developed. We will validate the model with other datasets to evaluate its generalizability. We are also following up a part of patients and plan on adding microalbuminuria and other new parameters through a large external cohort to verify the accuracy of the machine learning model. In addition, disease prediction models trained by longitudinal data may predict long-term CVD risks, to guide CVD prevention.

## Data availability statement

The original contributions presented in this study are included in the article/supplementary material, further inquiries can be directed to the corresponding author.

## Ethics statement

The studies involving human participants were reviewed and approved by Ethical Committee of Peking University People’s Hospital. The patients/participants provided their written informed consent to participate in this study.

## Author contributions

All authors listed have made a substantial, direct, and intellectual contribution to the work, and approved it for publication.
